# Oral hygiene and oral health behavior, periodontal complaints and oral health-related quality of life in pregnant women

**DOI:** 10.1186/s12903-022-02508-4

**Published:** 2022-11-08

**Authors:** Uwe Schröter, Dirk Ziebolz, Holger Stepan, Gerhard Schmalz

**Affiliations:** 1 Department of Cariology, Endodontology and Periodontology, University Medical Center Leipzig, Liebigstr. 10-14, D 04103 Leipzig, Germany; 2 Department of Obstetrics and Gynecology, University of Leipzig Faculty of Medicine, Leipzig, Germany

**Keywords:** Oral health-related quality of life, Pregnancy, Oral health behavior, Periodontal

## Abstract

**Objectives:**

This questionnaire-based cross-sectional study aimed in the evaluation of oral hygiene and oral health behavior, periodontal complaints and oral health-related quality of life (OHRQoL) in pregnant women in southwest-Saxony, Germany.

**Materials and methods:**

Consecutive patients attending the clinics for Obstetrics and Gynecology, Heinrich-Braun-Klinikum Zwickau, Germany, were recruited in the years 2020 and 2021. The evaluation consisted of three parts: (I) dental and oral hygiene behavior, (II) periodontal complaints and (III) German short form of oral health impact profile (OHIP G14) to assess OHRQoL.

**Results:**

853 out of 1056 participants were included in the study. The pregnant women reported that they have received information on oral health during pregnancy more often from gynecologists than from dentists. Slightly more than half of the participants (51.5%) rated to regularly undergo a professional tooth cleaning. Similarly, nearly half of the women stated to perform interdental cleaning (55.8%). The most common periodontal complaint was bleeding of the gums (45.4%). The OHIP G14 findings of all questions as well as sum scores showed a median of 0. Regression analysis revealed that regular professional tooth cleaning was a predictor of better OHRQoL (β – 0.698, CI95 0.049–1.299; p < 0.04).

**Conclusion:**

Oral hygiene and oral health behavior of pregnant women in southwest-Saxony requires improvement. While the overall OHRQoL of the cohort was not reduced, professional tooth cleaning and thus professional preventive measures can support OHRQoL during pregnancy. Improved interdisciplinary oral health care concepts for pregnant women should be fostered. These concepts can also positively influence OHRQoL issues.

## Introduction

Oral health, i.e., the absence of oral diseases as well as complaints related to teeth, gums, oral mucosa, temporomandibular joints and dentures is an important issue for systemic health and quality of life [1, 2]. Thereby, pregnancy is a period of particular vulnerability, because many physiological changes in the body of pregnant women can also affect the oral cavity [3]. Accordingly, changes in metabolism and hormone system, alongside with changed lifestyle and nutritional behaviors often lead to gingival and mucosal changes [3]. This might also include a potential risk for the unborn child; although discussed controversially, microorganisms and pro-inflammatory cytokines can pass the placental barrier, potentially leading to complications and adverse pregnancy events [4]. Moreover, there is a relationship between oral health of mother and child, especially with regard to dental caries [5]. Thereby, it seems like there is a lack in oral health information of pregnant women, making an integration of dentistry in prenatal care recommendable [6]. Overall, assessment of the oral health behavior and related parameters as well as potential strategies to support oral health in pregnant women are issues of high clinical and scientific interest [7].

Additionally, available studies show a reduced oral health-related quality of life (OHRQoL) in pregnant women [8, 9]. The OHRQoL, which is one part of the general health-related quality of life, reflects four distinct dimensions, including oral function, psychosocial impact, oral pain and orofacial appearance [10]. In this respect, the literature is somewhat inconclusive: while oral changes related to pregnancy appear to affect OHRQoL, mainly the psychosocial dimension seems to be reduced [9]. Moreover, the topic gains complexity due to the potential influence of oral health behavior, hyperglycemia and oral hygiene behaviors during early pregnancy on OHRQoL of pregnant women [11]. Furthermore, the OHRQoL findings always require an interpretation considering the frame conditions in the region of assessment, including health system and cultural issues [12].

Taken together, oral hygiene and oral health behavior, oral complaints and OHRQoL of pregnant women appear an issue of certain clinical and scientifical importance. Thereby, the body of data remains inconclusive and heterogeneous, whereby German studies with a comprehensive assessment and high sample size are still missing. Therefore, this current study aimed in the evaluation of oral hygiene and oral health behavior, periodontal complaints and OHRQoL of pregnant women in southwest-Saxony, Germany. Thereby, as many individuals as possible should be included, aiming in recruitment of a cross-sectional cohort with different duration of pregnancy and age. Additionally, it should be assessed whether there would be influential factors on the OHRQoL of those individuals.

## Materials and methods

### Study design

This current study was a cross-sectional examination, which has been reviewed and approved by the ethics committee of the medical faculty of Leipzig University (534/19-ek). All participating individuals were informed verbally and in writing about the study and provided a written informed consent prior to participation.

### Patients

For this questionnaire-based evaluation, pregnant women were consecutively recruited during their appointments for prenatal diagnostics, prenatal class or in-patient stay in the clinic of gynecology and obstetrics, Heinrich-Braun-Klinikum Zwickau, Germany. The period of investigation was between January 2020 and December 2021. Inclusion criteria were age of at least 18 years and a pregnancy at the time of inclusion. Only insufficient German language skills, making answering of the questions impossible, were defined as an exclusion criterion.

Following information and consent, participants were asked to fill out three different questionnaires anonymously.

### Questionnaires

The questionnaire-based evaluation included three different parts: (I) dental and oral hygiene behavior, (II) periodontal complaints and (III) Oral health impact profile (OHIP G14) to assess OHRQoL. The questionnaires were completed by the pregnant women and subsequently collected by the responsible physician.

Questionnaire (I) was composed based on available questionnaires from the working group, which have been used in comparable investigations of patients with chronic diseases or conditions with relevance for oral health [13, 14]. Thereby, the questionnaire was modified with regard to pregnancy-related issues. This questionnaire included questions regarding oral hygiene measures, which are performed by the respective patients, as well as patients’ utilization of dental consultations and preventions measures (e.g. professional tooth cleaning). Questionnaire (II) was composed based on an available survey to evaluate periodontal complaints [15], which was translated into German language. This questionnaire included 12 periodontal complaints, e.g., bleeding gums, tooth mobility or bad taste. (III) To evaluate OHRQoL, the German short form of the Oral Health Impact Profile (OHIP G14) was used, which is a valid measurement in this respect [16–18]. Using OHIP G14, complaints, which patients perceived in the previous month, related with their teeth, mouth or dentures were assessed on a five-point scale between 0 “never” and 4 “very often”. Thus, a higher OHIP G14 reflects worse OHRQoL [16, 17]. Those questions included e.g. trouble in pronouncing words, pain or a less satisfying life. Moreover, four dimensions, i.e., oral function, psychosocial impact, oral pain and orofacial appearance were estimated for evaluation [10]. A difference in median values of at least two points was interpreted as clinically relevant [19], and German reference values were used for further interpretation of the findings [17].

### Statistics

All data were assessed and recorded in an Excel sheet. First, a descriptive analysis, evaluating means, standard deviation, median and percentages was performed. Subsequently, associations between OHIP G14 and age, pregnancy week and number of pregnancies was performed using Kruskal-Wallis-test. In case of significance, a post-hoc testing with Bonferrroni-correction was applied. Finally, a regression analysis of some general and oral-health related parameters as potential predictors for OHIP G14 values as the dependent variable was performed. The significance level was set at p < 0.05.

## Results

### Patients

During the examination period, 1056 pregnant women were asked for their participation, of which 853 agreed to participate (participation rate: 80.8%). The mean age of the cohort was 30.46 ± 5.40 years, whereby the majority of patients was either in second or third trimester (Table 1).

**Table 1:** Characteristics of included individuals

Parameter	Pregnant women (n = 853)
Age in years (mv ± sd) [median; IQR]	30.46 ± 5.40 [31; 27–34]
Pregnancy week (mv ± sd) [median; IQR]	27.00 ± 7.50 [24; 22–35]
Number of pregnancies (mv ± sd) [median; IQR]	2.05 ± 1.31 [2; 1–3]

Mv: mean value, sd: standard deviation, IQR: inter-quartil-range

### Oral hygiene and oral health behavior

Approximately two third of patients visited the dentist since the pregnancy began (65.1%). Patients received the information on the importance of oral health during pregnancy more often by the gynecologist than from the dentist. Slightly more than half of patients rated that they undergo a regular professional tooth cleaning (51.8%). Similarly, nearly half of the participants reported to perform regular interdental cleaning (55.8%, Table 2).

**Table 2:** Oral health and oral hygiene behavior in the cohort

Question		Participants [n = 853]
Visited dentist until pregnancy began		65.1%
Information on oral health issues during pregnancy by dentist		54.5%
Information on oral health issues during pregnancy by gynecologist		71.7%
Visited the dentist because of information by the gynecologist		22.7%
How important was a healthy oral status before pregnancy for you (0 = not at all, 10 = very important)		8.05 ± 3.05
How important is a healthy oral status since pregnancy for you (0 = not at all, 10 = very important)		8.15 ± 3.06
Regular professional tooth cleaning		51.8%
Professional tooth cleaning since pregnancy began		43%
Information about dental prevention in children		46%
Do you feel well educated with oral health during pregnancy?		92.8%
Tooth brush	Power	50.2%
	Manual	63.1%
Interdental cleaning		55.8%
Oral rinse		53.8%

### Periodontal complaints

The most common periodontal complaint was bleeding of the gums, which was reported by 45.4% of individuals. Moreover, approximately one third (35.3%) reported on sensitive gums. More severe periodontal complaints like recession or tooth loosening was rarely reported by the included pregnant women (Table 3).

**Table 3:** Results of the questionnaire regarding periodontal complaints

Parameter	Participants (n = 853)
Gum swelling	14.6%
Painful gums	11.7%
Sensitive gums	35.3%
Bleeding gums	45.4%
Gingival recession	6.2%
Sensitive teeth	16.4%
Tooth loosening	2.9%
Tooth migration	1.2%
Changes in occlusion	0.7%
Halitosis	9.6%
Bad taste	12.7%
Tooth pain	10%

### OHRQoL

Values of OHIP G14 are displayed in Table 4, showing that the median of all questions and sum scores was 0. Regarding potential associations, a significant relation between age and OHIP G14 was revealed (p < 0.01). In post-hoc testing, a significant difference in OHIP G14 sum score between patients with an age < 25 years (2.71 ± 4.18 [1]) compared to those with age < 34 years (2.42 ± 5.40 [0]) was found (p < 0.01; Table 5). With regard to the principle of minimal important difference [19], this difference was not clinically relevant.

**Table 4:** Results of the OHIP G14 questionnaire

Parameter	Participants (n = 853)
Trouble pronouncing	0.06 ± 0.33 [0]
Taste worsened	0.16 ± 0.54 [0]
Life less satisfactory	0.15 ± 0.51 [0]
Difficult to relax	0.35 ± 0.77 [0]
Feeling of tension	0.21 ± 0.60 [0]
Interrupting meals	0.10 ± 0.43 [0]
Uncomfortable eating	0.22 ± 0.63 [0]
Short tempered	0.22 ± 0.60 [0]
Difficult to perform daily jobs	0.15 ± 0.54 [0]
Unable to function	0.07 ± 0.36 [0]
Embarassed	0.12 ± 0.45 [0]
Diet unsatisfactory	0.14 ± 0.48 [0]
Pain	0.35 ± 0.71 [0]
Sense of uncertainty with teeth	0.24 ± 0.66 [0]
Domain psychosocial impact	0.22 ± 0.69[0]
Domain oral function	0.23 ± 0.74[0]
Domain orofacial appearance	0.44 ± 0.99 [0]
Domain orofacial pain	0.54 ± 1.11 [0]
Total sum	2.44 ± 4.74 [0]

**Table 5:** Associations between OHRQoL and selected pregnancy-related parameters

Parameter		OHIP G14 sum
Age	< 25 years	2.71 ± 4.18 [1]*
	25–34 years	2.40 ± 4.61 [0]
	> 34 years	2.42 ± 5.40 [0]*
	p-value	< 0.01
Pregnancy week	0–20	2.53 ± 5.07 [0]
	21–30	2.73 ± 5.11 [0]
	> 30	2.02 ± 4.01 [0]
	p-value	0.31
Number of pregnancies	1	2.44 ± 4.64 [0]
	2–3	2.46 ± 4.86 [0]
	> 3	2.50 ± 4.91 [0]
	p-value	0.94

*significant difference in post-hoc testing (p < 0.01)

The regression analysis showed that regular professional tooth cleaning was an independent predictor of better OHRQoL (β – 0.698, CI95 0.049–1.299; p < 0.04; Table 6).

**Table 6:** Results of the regression analysis of OHIP G14 sum score as the dependent variable for different pregnancy- and oral health-related predictors

Model		Beta	p-value	Confidence interval (95.0%)	
				Lower	Upper
1	(Constant)	4.010	< 0.01	1.810	6.210
	Pregnancy week	-0.041	0.079	-0.086	0.005
	Age (years)	-0.009	0.792	-0.075	0.057
	Number of pregnancies (n)	-0.004	0.975	-0.275	0.267
	Regular professional tooth cleaning	-0.760	0.031	-1.449	-0.072
	Interdental cleaning	-0.099	0.779	-0.789	0.591
	Visited dentist since pregnancy began	0.396	0.283	-0.327	1.118
2	(Constant)	4.011	< 0.01	1.813	6.209
	Pregnancy week	-0.041	0.078	-0.086	0.005
	Age (years)	-0.009	0.766	-0.071	0.052
	Regular professional tooth cleaning	-0.759	0.029	-1.442	-0.076
	Interdental cleaning	-0.098	0.779	-0.787	0.590
	Visited dentist since pregnancy began	0.395	0.283	-0.327	1.117
3	(Constant)	4.018	< 0.01	1.821	6.214
	Pregnancy week	-0.041	0.075	-0.086	0.004
	Age (years)	-0.010	0.738	-0.071	0.051
	Regular professional tooth cleaning	-0.778	0.023	-1.447	-0.109
	Visited dentist since pregnancy began	0.383	0.294	-0.333	1.099
4	(Constant)	3.716	< 0.01	2.423	5.009
	Pregnancy week	-0.041	0.073	-0.087	0.004
	Regular professional tooth cleaning	-0.791	0.020	-1.455	-0.127
	Visited dentist since pregnancy began	0.379	0.298	-0.336	1.095
5	(Constant)	3.769	< 0.01	2.479	5.058
	Pregnancy week	-0.035	0.113	-0.079	0.008
	Regular professional tooth cleaning	-0.729	0.029	-1.382	-0.075
6	(Constant)	2.799	< 0.01	2.329	3.270
	Regular professional tooth cleaning	-0.698	0.036	-1.352	-0.045

## Discussion

Several deficits were revealed in the cohort of the current study. Thereby, only half of patients visited the dental practice for regular professional tooth cleaning since pregnancy began. While nearly half of the pregnant women reported on bleeding gums, the overall OHRQoL was not reduced in the cohort. Regression analysis showed that regular professional tooth cleaning was a predictor for better OHRQoL in the current study.

The necessity of an appropriate information regarding the importance of oral health for pregnant women and thus an interdisciplinary care approach between dentistry and gynecology has been demanded in Germany more than 30 years ago [20]. Meanwhile, there appears a positive trend in this respect, although a lack in information on oral health issues in pregnancy appears still present; a German questionnaire-based study of 83 pregnant women showed that only one quarter of patients was informed on oral health issues [21]. The current study found much higher values, whereby 54.5% (by dentist) and 71.7% (by gynecologist) rated that they were informed, while the vast majority felt well educated on oral health during pregnancy. The international literature regarding comparable questionnaire-based examinations showed heterogeneous findings, which also indicated a somewhat worse situation than in the current study [22–25]. A multicentric study in US showed an insufficient knowledge on oral health issues in pregnant women [22]. Similarly, two Italian studies showed an underestimation of oral health during pregnancy [23, 24]. An English survey pointed on a reduced oral health behavior in pregnant women [25]. Although the current study showed some deficits or needs of improvement, half of the participants in the current study used interdental cleaning and/or went to regular professional tooth cleaning, what appears to be slightly above average compared with the international literature. A qualitative Chinese study investigated potential barriers for pregnant women regarding their oral health behavior, whereby a lack or contradictory sources of information were the main issue [6]. These barriers seem to be less important in the current southern-Saxonian cohort; however, based on the current study´s results, there appears to be still need of improvement.

The current questionnaire-based survey considered periodontal complaints, but clinical examinations were not performed in the patient cohort. Thus, the real clinical oral health conditions of the participating individuals in the current study remain unknown and speculative. The respective age group, i.e. patients < 35 years is still not present in the population representative fifth German oral health study [26]; accordingly, reference values for the general population in this age group are missing. Independently, it is known that pregnant women often suffer from gingival inflammation [27]. This was also observed in another German study, which has been performed in Thuringia, what is near to southwest-Saxony [28]. Although the underlying etiology is not fully understood, yet, increased estrogen and progesterone during pregnancy have an immunological effect on gingival and periodontal tissues [27, 29]. Accordingly, the high prevalence of bleeding gums as an indicator for gingival inflammation in the current study appears plausible. Besides of the mainly reversible (pregnancy-)gingivitis, also more profound periodontal diseases can be related to pregnancy [30, 31]. This is comparatively rare, what explains the low prevalence of periodontitis-related complaints in the current study, including tooth loosening or bad taste. Against the background of the high prevalence of periodontal complaints (especially bleeding and sensitive gums), however, the OHRQoL of the cohort appears an issue of particular interest, and will be discussed in the following.

The OHRQoL is a multidimensional construct, illustrating the influence of oral health conditions on health-related quality of life [32]. Thereby, the OHIP G14 is a recommendable and appropriately validated measure for the assessment of OHRQoL, especially in clinical studies [32]. For interpretation of the values, reference values for the healthy German general population were reported [17]. Thereby, fully dentate individuals should have a median sum score of 0 [17], what is found in the current study, showing an overall unaffected OHRQoL of the study cohort. The unaffected OHRQoL in pregnant women as revealed in the current study, is contradictory to the available literature, whereby systematic reviews reported a worse or reduced OHRQoL in pregnancy, respectively [8, 9]. Thereby, main reasons for the reduced OHRQoL were oral complaints, especially in context of gingival inflammation [8, 9]. In detail, he four dimensions oral function, psychosocial impact, orofacial appearance and orofacial pain were estimated [10], whereby the dimension orofacial pain had a slightly higher mean value than the other ones. However, the median of all domains was zero and the differences appear not clinically relevant. Altogether, the findings of this study did not support an influence of oral complaints on OHRQoL in pregnant women. However, it is known that international and intercultural differences in the perception of OHRQoL exist [12], what limits the comparability of the local findings in this current monocentric survey. Regardless, two different explanations can be hypothesized for the OHRQoL findings in the current study: (I) the pregnant women in southwest-Saxony receive an appropriate oral health care and show (mainly) stable oral conditions. In the absence of a clinical oral examination, this hypothesis remains speculative. Nevertheless, the limited perception of (professional) dental prevention and the high prevalence of signs of gingival inflammation would argue against this explanation model. (II) Potentially, a shift in perception of the oral health situation in context of pregnancy appears possible, what might limit the extend, in which oral conditions are experienced as disturbing. This phenomenon has been described for chronically diseased individuals [33] and would fit better to the limited oral hygiene and oral health behavior of the cohort. For sure, comparing pregnancy with a chronic diseases is a very limited model. Moreover, in the absence of longitudinal as well as clinical data, this also remains speculation.

The regression analysis revealed that regular professional tooth cleaning was an independent predictor of OHRQoL. This would argue for a positive effect of prevention-oriented dental behavior to support OHRQoL during pregnancy. Because there is a lack of comparable literature in this respect, the transferability of those findings remain unclear. However, as a practical implication form the current results, the OHRQoL of pregnant in southwest-Saxony was found to be above average in international comparison, but improved dental care appear recommendable for those individuals. Thereby, increased sensibilisation and information, especially by the respective family dentists should be fostered.

This current study has its main strength in the large sample size, which is higher than in comparable national and international studies [20–25]. Moreover, the comprehensive use of validated questionnaire-based instruments revealed some new insights into the topic. Several limitations still require consideration. The monocentric character limits the ability to generalize the findings for the German general population. In addition, the absence of clinical examinations does not allow any conclusions on the real clinical situation of the patients. The survey was performed anonymously, without the possibility of any follow-up examinations, which would be of high interest in context of adverse outcomes and potential changes during and after pregnancy. Moreover, the level of education and sociodemographic parameters were not considered in the current study. Those parameters would have been of certain interest and should be recognized in future research projects. Therefore, several questions remain unacknowledged and should be recognized in the field.

### Conclusion

Oral hygiene and oral health behavior of pregnant women in southwest-Saxony requires some improvements. Additionally, gingival bleeding as a sign of gingival inflammation is a common complaint in those patients. While the OHRQoL in the overall cohort appear unaffected, professional preventive measures are a predictor of better OHRQoL in pregnant women. Therefore, interdisciplinary, prevention-oriented dental care should be fostered for pregnant women.

## Data Availability

The datasets used and/or analyzed during the current study are available from the corresponding author on reasonable request. The data are not publically available, because of the psedonymisation and data protection guidelines according to the ethics approval.
